# FAT4 Mutation is Related to Tumor Mutation Burden and Favorable Prognosis in Gastric Cancer

**DOI:** 10.2174/0113892029300694240612081006

**Published:** 2024-06-14

**Authors:** Qingqing Li, Yuxin Chu, Yi Yao, Qibin Song

**Affiliations:** 1 Cancer Center, Renmin Hospital of Wuhan University, Wuhan, 430060, China

**Keywords:** Gastric cancer, FAT4, tumor mutation burden, prognosis, somatic mutation data of GC, tailored therapy

## Abstract

**Objective:**

This study aimed to investigate the frequently mutated genes in Gastric Cancer (GC), assess their association with Tumor Mutation Burden (TMB) and the patients’ survival, and identify the potential biomarkers for tailored therapy.

**Methods:**

Simple somatic mutation data of GC were collected from the TCGA and ICGC databases. The high-frequency mutated genes were identified from both datasets. The samples were initially dichotomized into wild-type and mutation groups based on the status of overlapping genes. TMB difference between the two groups was evaluated by the Mann-Whitney U-test. Survival difference between the two groups was compared by the Kaplan-Meier method with a log-rank test. The prognostic value of the target gene was assessed by the Cox proportional hazards model. The signaling pathways involved in FAT4 mutation were identified by Gene Set Enrichment Analysis (GSEA). The fractions of different tumor-infiltrating immune cells were calculated by the CIBERSORT algorithm.

**Results:**

21 overlapping genes with frequent mutation were identified in both datasets. Mutation of these genes was significantly associated with higher TMB (*P*<0.05) in GC. The survival of the FAT4 mutation group was superior to the wild-type group. FAT4 mutation was also identified as an independent favorable prognostic factor for the GC patients. GSEA indicated that FAT4 mutation activated the signaling pathways involved in energy metabolism. Finally, CD4 memory-activated T cells, follicular helper T cells, and gamma delta T cells were significantly more enriched, while naïve B cells and regulatory T cells (Tregs) were significantly less enriched in the FAT4 mutation group (*P*<0.05).

**Conclusion:**

FAT4 mutation is relevant to TMB and favorable prognosis in GC, which may become a useful biomarker for immunotherapy of GC patients.

## 1 INTRODUCTION

Gastric Cancer (GC) is a prevalent cancer worldwide. In light of the latest global cancer statistics, over one million new cases of GC were diagnosed in 2020, leading to an estimated 769,000 deaths [1]. GC has ranked fifth for incidence and fourth for mortality in the world [1]. In Asia, the morbidity of GC accounts for approximately 60% of the cases, posing a big health problem [2]. Currently, the survival of these patients is still unsatisfactory. Hence, it is urgent to find new biomarkers to optimize prognosis prediction and achieve more individualized treatment.

Tumor Mutation Burden (TMB) has been reported to predict tumor behavior and immune response [3]. Tumors with non-synonymous mutations are liable to produce new antigens, which may enhance the immunogenicity of cancer [4]. Cancer cells with high TMB can be more easily recognized by the immune system, and stimulate stronger anti-tumor immune response [5]. Patients with high TMB have a significantly superior response to immune checkpoint inhibitors [6]. Recent studies have explored the somatic mutation landscape in gastric cancer using next-generation sequencing approaches. For instance, a study used a median sequencing coverage depth of 708-fold, revealing Tumor Protein P53 (TP53) gene mutation as the most frequent genetic alteration in GC [7]. Another study identified new significantly mutated driver genes in GC, such as MUC6, CTNNA2, GLI3, RNF43, and others [8]. Mechanistically, a similar study reported somatic mutations in the WNT, Hedgehog, cell cycle, DNA damage, and Epithelial-to-mesenchymal Transition (EMT) pathways in GC [9]. The identified novel mutations may affect clinical response and direct new targets for drug discovery. However, the relationship between specific gene mutations and TMB, as well as their prognostic implications, remains to be further elucidated. FAT atypical cadherin 4 (FAT4) is a cancer suppressor that was initially identified in mouse mammary epithelial cells [10]. FAT4 gene mutation has been identified in 5% of GC patients, and is more common in intestinal-type GC [11]. FAT4 has been reported to inhibit EMT and promote autophagy in colorectal cancer cells [12]. Nevertheless, the relationship between FAT4 mutation and TMB in GC has not been reported yet, nor the prognostic value of FAT4 mutation for GC patients.

In this study, we employed a systems biology approach to investigate the frequently mutated genes in GC and their association with TMB and patient survival. Systems biology approaches integrating multi-omics data and computational methods have been widely employed to investigate complex biological processes and identify potential biomarkers in cancer research [13]. The computational techniques employed in systems biology offer a comprehensive understanding of interactions and dynamics across multiple scales [14]. We utilized bioinformatics tools and algorithms to analyze large-scale genomic data from public databases and detect mutated genes associated with TMB and clinical outcomes in GC. We aimed to identify FAT4 as a useful biomarker for predicting TMB and prognosis of the GC patients. Our study will hopefully improve the efficacy of immunotherapy for these patients.

## 2 MATERIALS AND METHODS

### 2.1 Data Source

The transcriptome (FPKM value), somatic mutation, and clinical data of stomach cancer were extracted from the TCGA database (https://portal.gdc.cancer.gov/repository). Meanwhile, the simple somatic mutation data in Japanese patients were downloaded from the ICGC database (https://dcc.icgc.org/releases/current/Projects/GACA-JP). The fragmented data in multiple files were integrated into one file with a Perl program for subsequent analysis in R software. We excluded the cases with incomplete clinical data, such as age, gender, TNM stage, follow-up time, and survival status.

### 2.2 Identification of Top Mutated Genes

The somatic mutations were analyzed using the 'GenVisR' package in R software, which employs a matrix visualization algorithm to display genomic data in a compact and intuitive manner. The .maf files with somatic mutation information of the TCGA cohort were visualized in waterfall pictures by the “GenVisR” package. The gene variants in the .tsv file of the ICGC cohort were annotated in terms of the human reference genome. In much the same way, the somatic variants in GC samples of the ICGC cohort have also been displayed in the waterfall plot. The top 30 frequently mutated genes were identified in both cohorts. The common mutated genes were acquired by intersecting the top 30 mutated genes in GC between the TCGA and ICGC cohorts.

### 2.3 Defining TMB and Performing Survival Analysis

TMB was defined as the number of non-synonymous mutations (base substitutions, insertions/deletions) per megabase of the coding genome, as described in the harmonization efforts [15]. We only counted the gene mutations that could engender the changes in amino acids. The TMB value of every GC sample in the TCGA database could be calculated according to this TMB formula [16]. The correlation between TMB and the status of common mutated genes was evaluated by the “ggpubr” R package. The survival impact of each gene mutation was assessed by the Kaplan-Meier method with a log-rank test. The significant gene was further included in Cox regression models to prove the prognostic value.

### 2.4 Gene Set Enrichment Analysis

The RNA-seq data of GC samples were extracted from the TCGA database. The samples were dichotomized into the mutation group and wild-type group based on the mutation status of the target gene. We performed Gene Set Enrichment Analysis (GSEA) to find the signaling pathways significantly affected by FAT4 mutation. We adopted the “c2.cp.kegg.v7.1.symbols.gmt” as a reference gene set from the MsigDB database.

### 2.5 Tumor-infiltrating Immune Cell Analysis

The fraction of tumor-infiltrating immune cells was calculated using the CIBERSORT algorithm, which applies a machine learning approach to deconvolute gene expression profiles and estimate the relative abundance of different immune cell subsets. We set *P*<0.05 as a threshold to filter the immune cell subsets. The samples were divided into 2 groups in light of FAT4 mutation status. We compared the relative abundance of various immune cells in each group. The correlation between the fraction of immune cells and target gene status was visualized by violin plots.

### 2.6 Statistical Analysis

The association of TMB with gene mutation status was evaluated by the Mann-Whitney U test [5]. Survival analyses were conducted by employing the Kaplan-Meier method and Cox proportional hazards models. The correlation between gene mutation status and tumor-infiltrating immune cells was analyzed by Wilcoxon signed-rank test. A two-tailed *P*<0.05 indicated statistical significance.

## 3 RESULTS

### 3.1 The Somatic Mutation Landscape of GC in TCGA and ICGC Cohorts

**Fig. (1):**
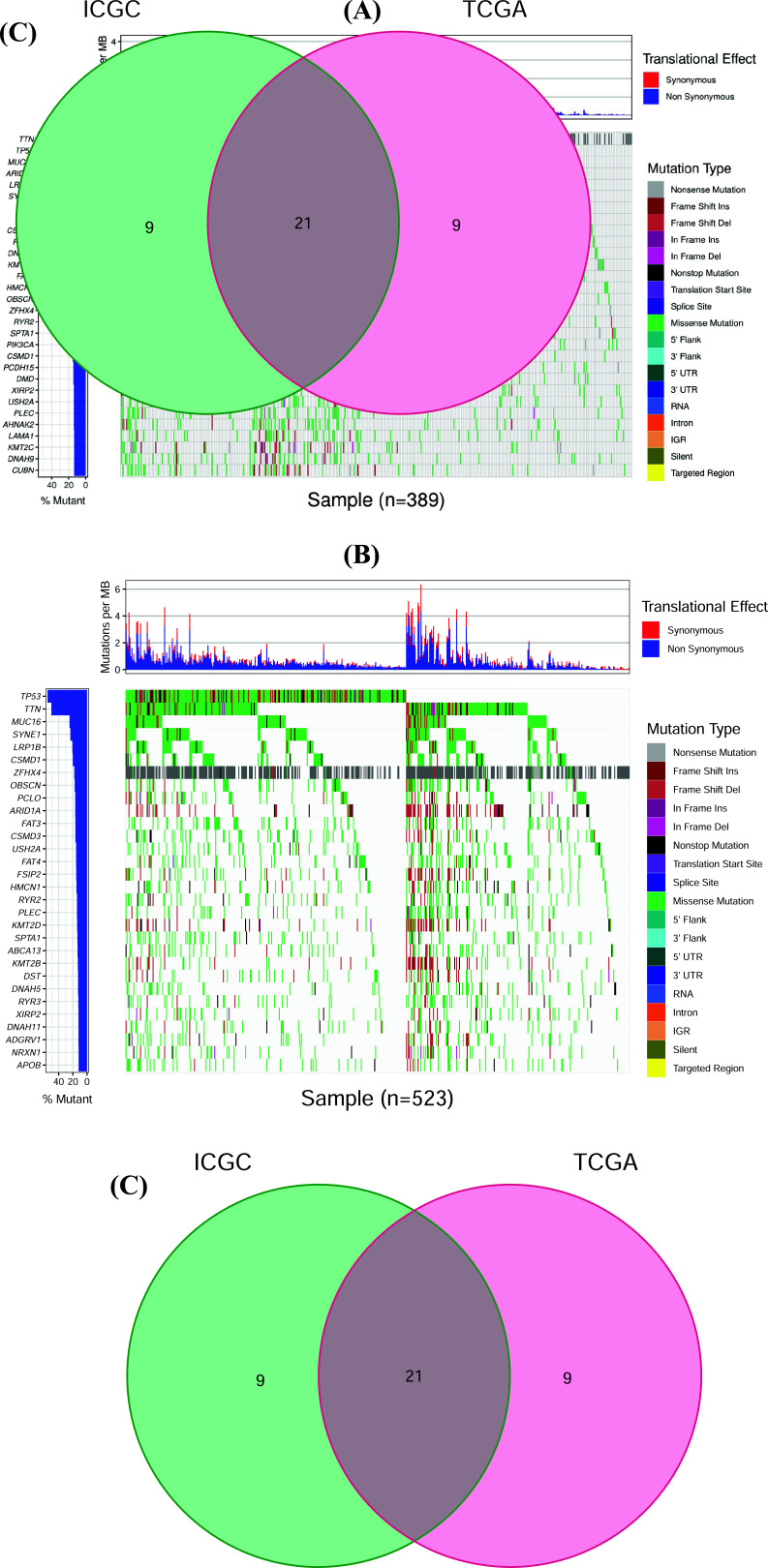
The gene mutation landscape in GC. (**A**) The waterfall picture shows the top 30 genes with frequent mutations in the TCGA cohort. (**B**) The waterfall plot indicates the top 30 mutated genes in the ICGC cohort. The top barplot represents the TMB in each sample. The left barplot reflects the mutation frequency of each gene. Different colors represent the mutation types in GC samples. (**C**) The Venn diagram indicates the number of frequent and common mutated genes in TCGA and ICGC cohorts.

We collected 389 samples from the TCGA database and 523 samples from the ICGC database. Then we analyzed the somatic mutation pattern of the top 30 high-frequency mutant genes in the TCGA cohort. The commonest variant was the missense mutation. The five highest frequently mutated genes were TTN, TP53, MUC16, ARID1A, and LRP1B (Fig. **1A**). In parallel, we also explored the somatic mutation spectrum of the top 30 frequently mutated genes in the ICGC cohort. Among them, the top 5 genes were TP53, TTN, MUC16, SYNE1, and LRP1B (Fig. **1B**). Furthermore, we screened 21 overlapping genes with the highest mutation frequency in GC from both databases by a Venn diagram (Fig. **1C**). These commonly mutated genes have been recruited for subsequent analysis.

### 3.2 Association between Gene Mutation and TMB in GC

**Fig. (2):**
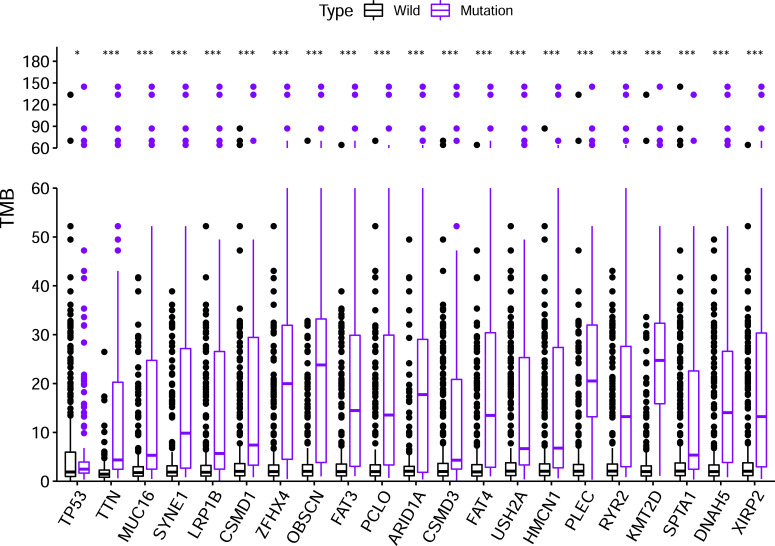
Relationship between gene mutations and TMB. The mutation type of all the 21 overlapping genes has been significantly related to an elevated TMB.

To further explore the association between gene mutation and TMB, we computed the TMB value of each sample in the TCGA cohort. According to the mutation status of 21 overlapping genes, the samples were divided into the wild-type group and the mutation group. Compared to the wild-type group, all the genes had significantly higher TMB in the mutation group of GC samples (all *P*<0.05, Fig. **2**).

### 3.3 Survival Analysis

**Fig. (3):**
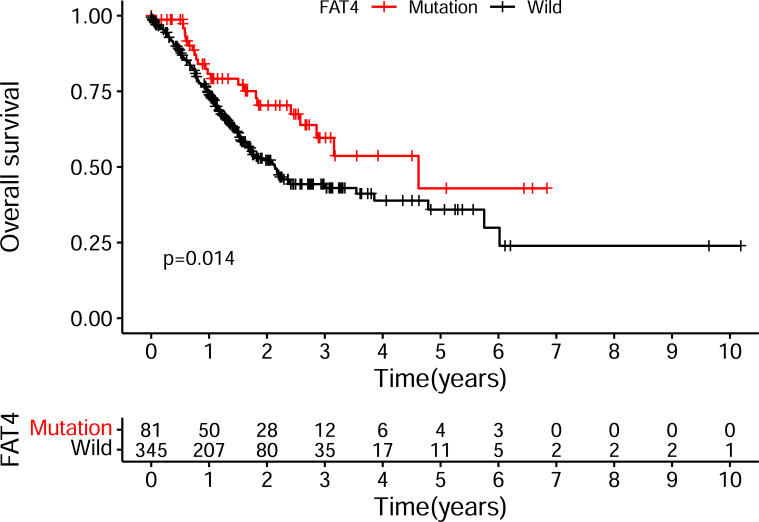
FAT4 mutation is significantly associated with the overall survival of GC patients by Kaplan-Meier curves (*P*<0.05).

To investigate the survival impact of 21 intersecting gene mutations, we performed the Kaplan-Meier survival analysis. We have found only FAT4 mutation to be significantly correlated with the Overall Survival (OS) of patients (Fig. **3**, *P*<0.05). Specifically, the group of patients with FAT4 mutation presented a superior survival to the wild-type group. Survival analysis by mutation status of other genes did not show statistical significance. As a result, we regarded FAT4 as our target gene for the subsequent analysis.

### 3.4 The Prognostic Value of FAT4 Mutation in GC

**Fig. (4):**
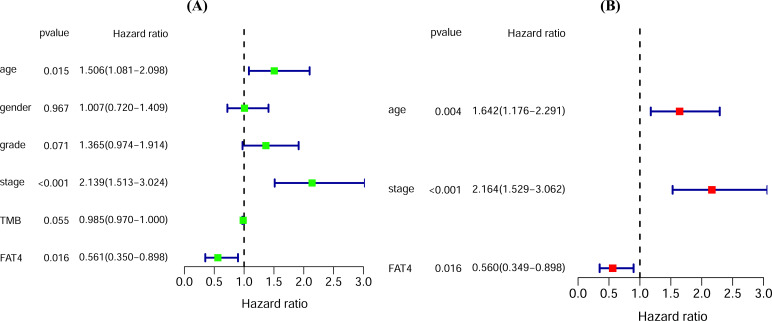
The forest plots indicating the results of Cox regression analysis. (**A**) Univariate Cox regression analysis. (**B**) Multivariate Cox regression analysis after adjusting confounders.

In order to evaluate the prognostic value of FAT4 mutation in GC, the mutation status of FAT4 along with clinical factors, such as age, sex, grade, TNM stage, and TMB, were inputted to the Cox regression models. In univariate COX regression analysis, we found age, stage, and FAT4 mutation to be significant prognostic indicators (Fig. **4A**, *P*<0.05). In the multivariate COX regression model, age, stage, and FAT4 mutation status were significantly associated with the patients’ survival (Fig. **4B**, *P*<0.05). Therefore, FAT4 mutation (HR<1, *P*<0.05) was identified as an independent favorable prognostic factor for the GC patients.

### 3.5 Enrichment of Pathways for Patients with FAT4 Mutation

**Fig. (5):**
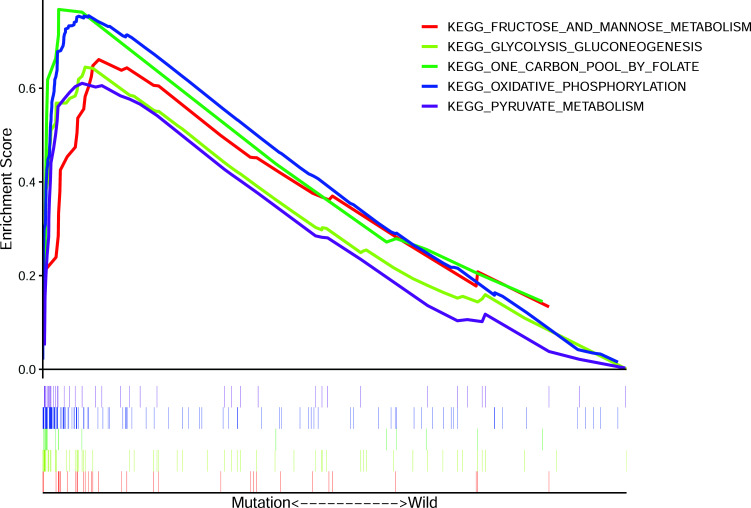
GSEA reveals significantly activated pathways related to FAT4 mutation. Specifically, FAT4 mutation is correlated with multiple pathways involved in energy metabolism.

We adopted GSEA to investigate the functional role of FTA4 mutation in GC samples from the TCGA cohort. The results demonstrated oxidative phosphorylation, glycolysis gluconeogenesis, one carbon pool by folate, fructose and mannose metabolism, and pyruvate metabolism to be significantly enriched in FTA4 mutation samples (Fig. **5**). These results imply that FTA4 mutation may activate the signaling pathways relevant to energy metabolism, leading to the occurrence of GC.

### 3.6 Tumor-infiltrating Immune Cells Relevant to FAT4 Mutation in GC

**Fig. (6):**
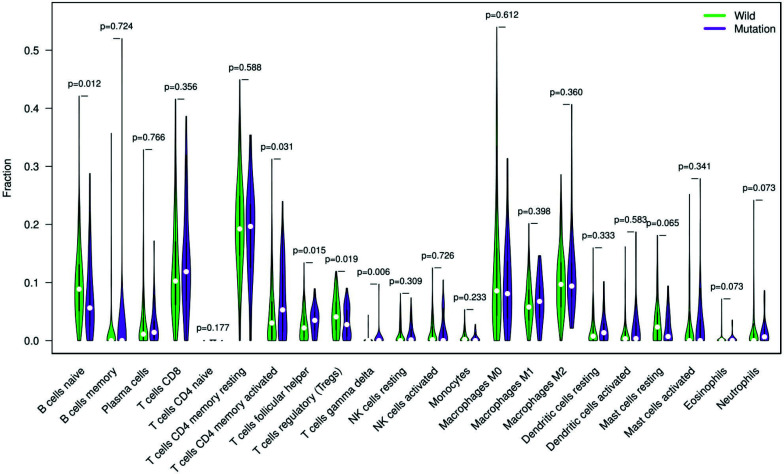
Violin plot reflects the different fractions of tumor-infiltrating immune cells in the FAT4-wild group *versus* the FAT4-mutation group. Green color represents the FAT4-wildtype group, while purple denotes the FAT4-mutation group.

To further explore the influence of FAT4 mutation on the abundance of different tumor-infiltrating immune cells, we employed the CIBERSORT algorithm to estimate the fraction of 22 immune cells in each tumor sample. The violin plot indicated CD4 memory-activated T cells, follicular helper T cells, and gamma delta T cells to be significantly more enriched in the FAT4 mutation group. In contrast, naïve B cells and regulatory T cells (Tregs) were significantly less enriched in the FAT4 mutation group (Fig. **6**, *P*<0.05).

## 4 DISCUSSION

Our study has analyzed the somatic mutation data of GC samples from the TCGA and ICGC databases. We have found the mutation type of all 21 genes to be significantly associated with higher TMB than the wild type. FAT4 was identified as the only significant gene by KM survival analysis. Patients with FAT4 mutation showed a significantly superior survival to the wild-type counterpart. Additionally, FAT4 mutation was positively related to the signaling pathways in energy metabolism. Finally, FAT4 mutation samples implicated more infiltration of CD4 memory-activated T cells, follicular helper T cells, and gamma delta T cells, while less infiltration of naïve B cells and Tregs. These variations may play a vital role in the GC immune microenvironment.

Previous studies have investigated the associations between individual gene mutations and TMB or clinical outcomes in GC. For example, a recent study identified SCN7A as a key TMB-related gene in GC; the expression of SCN7A in tumor tissues was lower than that in normal tissues, and low SCN7A expression was relevant to better prognosis [17]. Another study identified PTCH1 mutation as a potential predictive biomarker for immune checkpoint inhibitors in gastrointestinal cancer; the PTCH1-mutation group showed significantly better survival, higher TMB, and higher expression of most immune-related genes [18]. An additional study reported MUC16 as one of the most frequently mutated genes in patients with GC. MUC16 mutation was linked to better prognosis, including lower LNM rates and improved survival rates [19]. Moreover, MUC16 mutation status was correlated with TMB and MSI statuses [19]. Comparatively, our study has adopted a comprehensive approach by initially screening frequently mutated genes across multiple cohorts, which has been followed by a detailed analysis of the top candidate gene FAT4 in terms of its relationship with TMB, prognosis, signaling pathways, and tumor immune microenvironment. Our findings provide novel insights into the biological and clinical implications of FAT4 mutation in this malignancy. The multi-faceted analysis has provided a more comprehensive characterization of the potential utility of FAT4 mutation as a biomarker in GC.

FAT4 has been reported to maintain planar cell polarity and inhibit cell proliferation [20]. FAT4 gene mutation is also recurrent in many human cancers. Owing to hypermethylation in the promoter of gene, FAT4 expression is suppressed in lung cancer, enhancing its malignancy [21]. A missense mutation of FAT4 was also identified in peritoneal metastatic pancreatic cancer [22]. Moreover, FAT4 mutation could compromise the function of repressing cell proliferation, invasion, and metastasis in GC [23]. Our study has also identified FAT4 as a highly frequently mutated gene in GC. We have found FAT4 mutation to be positively associated with TMB. Our findings are consistent with a recent study that has revealed patients with high TMB to have better survival outcomes in GC, which might promote immune infiltration and provide new ideas for immunotherapy [24]. Therefore, FAT4 mutation may reflect high TMB and better prognosis of GC patients.

While TMB has emerged as a promising biomarker for predicting immunotherapy response, its quantification can be influenced by various factors, including the sequencing platform and bioinformatics pipelines used [25]. Additionally, TMB reflects the overall mutational load, but does not provide insights into specific mutated genes that may contribute to tumor behavior and immune response [26]. In this study, we have identified frequently mutated individual genes associated with TMB and clinical outcomes in GC, which could potentially serve as complementary biomarkers alongside TMB. Specifically, we have focused on FAT4, a tumor suppressor gene, as our analysis has revealed its mutation status to be significantly correlated with TMB and prognosis in GC patients. We have used a definition of TMB consistent with efforts of the TMB Harmonization Project.

With respect to the prognostic value of FAT4 mutation in GC, a recent study has explored the role of FAT4 in GC progression. It demonstrated low FAT4 expression as associated with poor survival in GC patients [27]. Another study has investigated the consequence of losing FAT4 expression and its correlation with clinical risk factors in radically resected GC [28]. It has revealed the loss of FAT4 expression to lead to increased invasion of GC, leading to an unfavorable prognosis. Comparatively, our study has indicated FAT4 mutation to reflect better survival than the wild type. FAT4 mutation has been identified as an independent favorable prognostic factor for GC patients. FAT4 mutation has been found to be significantly correlated with high TMB in GC samples, which may facilitate the exposure of neoantigens to the immune system, eliciting an enhanced anti-tumor immune response. Thus, we speculate that the superior survival in the FAT4 mutation group may be attributed to the anti-tumor immunity induced by high TMB.

As for the signaling pathways involving FAT4 in cancer, a recent study has reported FAT4 silencing to enhance EMT and invasiveness of ovarian cancer by regulating YAP and β-catenin activity [29]. Additionally, another study showed that miR-107 suppressed FAT4 expression and regulated the invasion of GC cells by activating the PI3K-AKT signaling pathway [30]. In GSEA, we have found FAT4 mutation to enhance the activity of signaling pathways involved in energy metabolism in GC. In line with our findings, a recent study has established a prognostic signature in terms of energy metabolism-related genes in stomach cancer [31]. It showed high-risk GC to be implicated in ECM receptor interaction and hedgehog signaling pathway. These identified pathways may become useful for interpreting the role of FAT4 mutation in the development of GC.

In tumor-infiltrating immune analysis, we have discovered FAT4 mutation to involve more infiltration of CD4 memory-activated T cells, follicular helper T cells, and gamma delta T cells, while less infiltration of naïve B cells and Tregs. Increased CD4+ memory T cells have been detected in tumor-draining lymph nodes of breast cancer patients [32]. These memory T cells can generate an immune response against tumor antigens and prevent the relapse of cancer, leading to prolonged survival. On the other hand, the faction of Tregs was lower in the FAT4 mutation group than the wild type in GC. It is well known that Tregs function in maintaining self-tolerance and limiting excessive immune response [33]. We suppose that FAT4 mutation decreased Tregs infiltration, engendering enhanced antitumor immunity. Therefore, FAT4 mutation may promote anti-tumor immunity by inducing the variation of tumor-infiltrating immune cells in GC.

Several limitations of the study should be noted. First, it is important to note that mutation frequency is a confounded measure, as it is influenced by both the underlying mutation rate and the selective advantage conferred by the mutation in cancer development [34]. Our analysis based on mutation frequency may not have fully captured the functional significance of the identified genes in GC progression. Further analysis is required to differentiate the contributing factors for individual genes. Second, our study has been based on pure bioinformatics analysis; thus, future experiments are necessary to validate our findings. Third, while our study has demonstrated the association between FAT4 mutation and increased TMB, further analysis is needed to differentiate the underlying mutation rate and selective advantage conferred by FAT4 mutation in GC development [35]. Such an analysis would help quantify whether FAT4 is a major driver, a minor driver, or merely a neutral proxy for mutation burden compared to other frequently mutated genes in GC. This differentiation is crucial for determining the functional significance of FAT4 mutation in tumor biology and its potential as a therapeutic target.

## CONCLUSION

FAT4 mutation is frequent and associated with higher TMB in gastric cancer, leading to an anti-tumor immune response. FAT4 mutation is also related to better survival than the wild type. The patients with FAT4 mutation may benefit from immunotherapy. Our study may facilitate the identification of specific patients for tailored treatment, and hopefully improve their clinical outcome.

## AUTHORS' CONTRIBUTIONS

Q. Li integrated the data and performed statistical analysis, Y. Chu drafted the manuscript, and Y. Yao and Q. Song supervised the study.

## Data Availability

Data are publicly available and accessible on TCGA (https://portal.gdc.cancer.gov) and ICGC (https://dcc.icgc.org) databases.

## LIST OF ABBREVIATIONS

EMT: Epithelial-to-mesenchymal Transition
GC: Gastric Cancer
GSEA: Gene Set Enrichment Analysis
OS: Overall Survival
TMB: Tumor Mutation Burden
